# Correlation Between Circulating PCSK9 Levels and Gestational Diabetes Mellitus in a Chinese Population

**DOI:** 10.3389/fendo.2022.826757

**Published:** 2022-04-13

**Authors:** Yiming Wu, Jie Shi, Qing Su, Zhen Yang, Li Qin

**Affiliations:** ^1^ Department of Endocrinology, Xinhua Hospital Chongming Branch, Shanghai Jiaotong University School of Medicine, Shanghai, China; ^2^ Department of Endocrinology, Xinhua Hospital, Shanghai Jiaotong University School of Medicine, Shanghai, China

**Keywords:** proprotein convertase subtilisin/kexin type 9 (PCSK9), gestational (gestational diabetes), lipid, glucose–insulin, metabolism

## Abstract

**Background:**

Previous studies reported that proprotein convertase subtilisin/kexin type 9 (PCSK9) was a key player in the regulations of lipid metabolism and glucose homeostasis. The current study aimed to detect the expression of PCSK9 in pregnant women with gestational diabetes mellitus (GDM) and investigate the possible relationships between PCSK9 and related metabolic phenotypes in GDM.

**Methods:**

Circulating PCSK9 levels were determined by ELISA kit in a cohort of subjects with GDM (n = 170) and normal glucose tolerance (NGT; n = 130). We collected blood samples from all participants for the biochemical index determinations. Diagnosis of GDM was made according to the International Association of the Diabetes and Pregnancy Study Groups Consensus Panel. Correlation analysis and logistic regression analysis were used to study the potential associations between PCSK9 and GDM.

**Results:**

GDM women presented significantly higher circulating PCSK9 levels than those in NGT pregnant subjects (268.07 ± 77.17 vs. 254.24 ± 74.22 ng/ml, P < 0.05). In the GDM group, serum PCSK9 levels were positively correlated with fasting plasma glucose (FPG) (R = 0.251, P = 0.015), glycated hemoglobin (HbA1c) (R = 0.275, P = 0.009), total cholesterol (TC) (R = 0.273, P = 0.010), and low-density lipoprotein cholesterol (LDL-C) (R = 0.326, P = 0.002) after adjustment of age and gestational age. Logistic regression found that age [odds ratio (OR) = 5.412, P = 0.02] and serum PCSK9 levels (OR = 4.696, P = 0.03) were independently associated with GDM. Compared with the lowest serum PCSK9 level quartile group, the prevalence of GDM was significantly higher in the highest quartile group, the ORs of GDM were 3.485 (95% CI 1.408–8.627, P < 0.05 for the trend), after adjusting for potential confounders.

**Conclusions:**

Circulating PCSK9 levels were associated with dyslipidemia, pathoglycemia, and the risk of incident GDM, indicating a potential link between PCSK9 and GDM.

## Introduction

The American Diabetes Association (ADA) defines gestational diabetes mellitus (GDM) as any degree of glucose intolerance that was first recognized during pregnancy, regardless of the degree of hyperglycemia (1). GDM is a common metabolic complication that develops as gestation proceeds. It contributes to adverse metabolic disorders including type 2 diabetes mellitus (T2DM), obesity, and metabolic syndrome both in mother and fetus later in life (2–4). In recent decades, the incidence of GDM continued to increase worldwide (5). According to a meta-analysis, the GDM prevalence in China was reported to be 11.91% (6). It should be noted that increased insulin resistance and the following β-cell dysfunction take part in the development of GDM, but the exact pathogenesis of GDM have not been fully understood yet (7).

Proprotein convertase subtilisin/kexin type 9 (PCSK9) is the ninth member of subtilisin-like serine convertase superfamily and mainly derived from the liver (8). It is a central regulator of low-density lipoprotein (LDL) receptor (LDL-R) expression, by promoting the clearance of LDL-R, resulting in subsequent increased plasma LDL cholesterol (LDL-C) levels and hypercholesterolemia (9). Despite the previously observed close associations of PCSK9 with dyslipidemia, it was reported that PCSK9 also have effects on other metabolic diseases, but the results were controversial. Previous data observed that PCSK9 levels were increased in type 2 diabetes mellitus/metabolic syndrome patients and positive correlations of PCSK9 levels with LDL, fasting plasma glucose (FPG), glycosylated hemoglobin (HbA1c), fasting insulin, and insulin resistance (10, 11). Moreover, a recent population-based longitudinal study observed a positive association between serum PCSK9 levels and the incidence of T2DM in the prediabetic populations (12). In contrast, evidence from an animal study indicated that PCSK9 deficiency reduced insulin secretion and promoted glucose intolerance (13). These studies suggested a key role for PCSK9 in the progression of DM.

Unlike other cytokines, circulating levels of PCSK9 in GDM subjects have been little studied and poorly understood until now. In view of the regulation effects of PCSK9 on lipid metabolism, we sought to investigate the plasma PCSK9 levels in GDM patients and its possible relationships with GDM.

## Materials and Methods

### Study Population and Design

**Figure 1:**
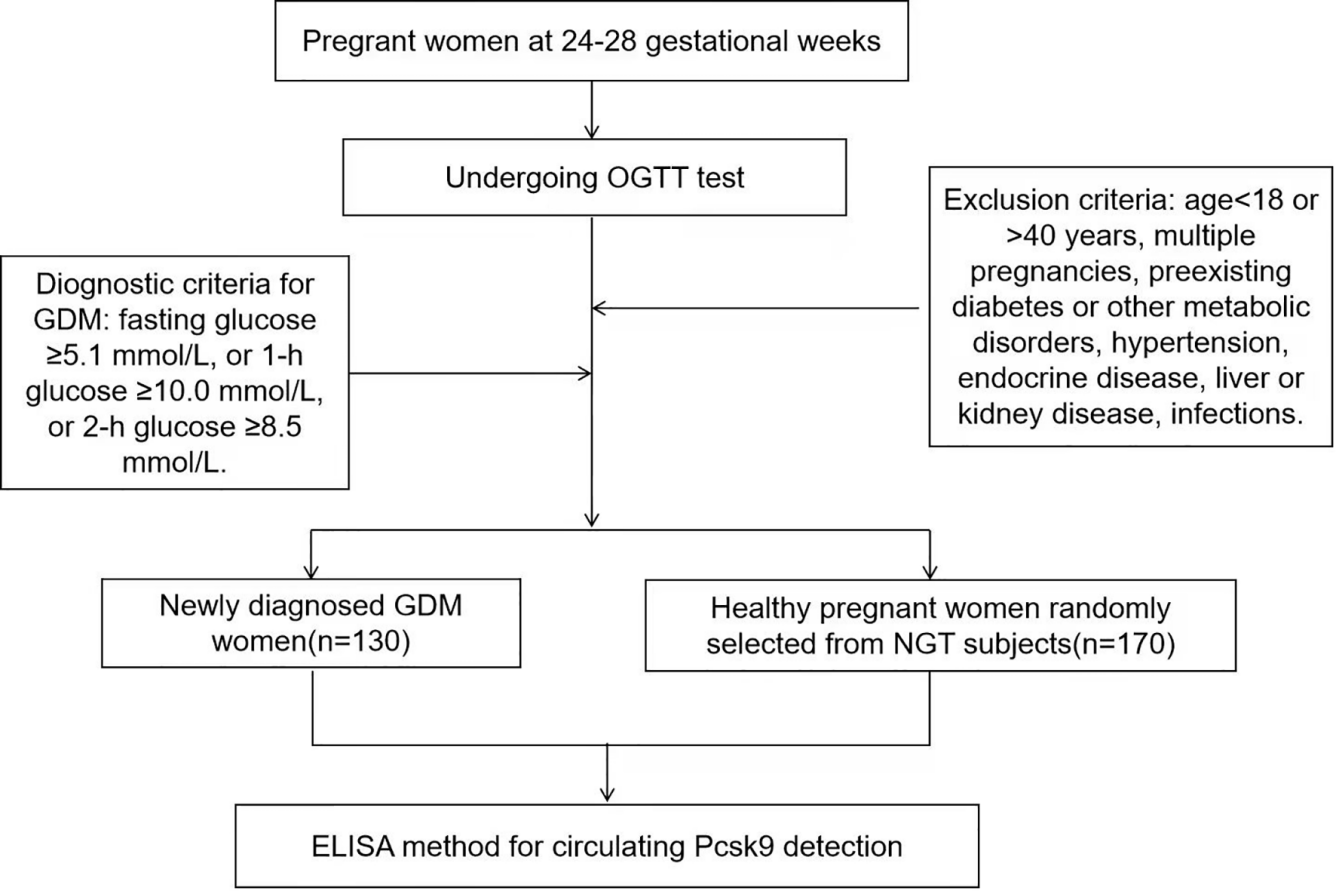
Flow graph of study design.

We recruited second-trimester pregnant women (gestational weeks 24–28) who attended an antenatal outpatient clinic in the Xinhua Hospital Chongming Branch Affiliated to Shanghai Jiao Tong University School of Medicine between January and December 2020. This cross-sectional study comprised 130 newly diagnosed GDM women (GDM group, n = 130) and 170 healthy pregnant women randomly selected from normal glucose tolerance (NGT) subjects (NGT group, n = 170) according to the random number. A total of 300 participants were enrolled. The exclusion criteria were as follows: age <18 or >40 years, multiple pregnancies, preexisting diabetes or other metabolic disorders, hypertension, endocrine disease, liver or kidney disease, infections. The study protocol was in compliance with the Declaration of Helsinki and approved by the ethics committee of the hospital. Each participant has read and written the informed consent. Study design of this current trial was described in **Figure 1**.

### Diagnosis of Gestational Diabetes Mellitus

All subjects underwent a 75-g oral glucose tolerance test (OGTT) screening for GDM during 24–28 gestational weeks. Using the criteria of the International Association of the Diabetes and Pregnancy Study Groups, GDM is diagnosed when any of the following plasma glucose values is met or exceeded: fasting glucose ≥5.1 mmol/L, 1-h glucose ≥10.0 mmol/L, or 2-h glucose ≥8.5 mmol/L (14).

### Clinical Characteristics and Laboratory Measurements

Basic information of name, age, pre-pregnancy weight, history of gravidity and parity, and family history of diseases was obtained using self-reported questionnaire from all subjects at the first prenatal examination during 13–15 gestational weeks. Anthropometric indices including height, weight, blood pressure, and abdominal girth were measured according to international standards in the second trimester. After overnight fasting, blood samples were collected during the course of OGTT that was undertaken between the 24th and 28th week of gestation. Then, the samples were separated and frozen at -80℃ for later analysis. Blood glucose levels, HbA1c, serum insulin levels, serum creatinine (Cr), triglyceride (TG), total cholesterol (TC), high-density lipoprotein cholesterol (HDL-C), LDL-C, alanine aminotransferase (ALT), aspartate transaminase (AST), γ-glutamyltransferase (GGT), and white blood cell count were detected by standard laboratory methods in the clinical laboratory of our hospital. Furthermore, the plasma PCSK9 values were measured by sandwich ELISA assay (R&D Systems, Minneapolis, MN, USA) based on manufacturer’s instruction. Each sample was detected in duplicate; the lowest limit of detection was 91 pg/ml with intra- and inter-assay coefficients of variation of 2.32%–8.91% and 4.54%–10.22%, respectively. Homeostasis model assessment of insulin resistance (HOMA-IR) was determined using the formula: HOMA-IR = fasting insulin (mU/L) × FPG (mmol/l)/22.5; HOMA-β assuming the pancreatic β-cells’ function was calculated as [FINS × 20/(FPG-3.5)] (15); insulin sensitivity was calculated by the Matsuda and de Fronzo index (ISOGTT), defined as [10,000/sqrt (FPG × FINS × mean glucose × mean insulin)].

### Statistical Analysis

We used Social Sciences software version 22.0 (SPSS, Chicago, IL, USA) for data analysis. Continuous variables were presented as mean ± SD or medians (interquartile range); for comparisons between groups, we used independent-samples t-test or Mann–Whitney U test based on different data distributions. Categorical variables were reported as rate (%), comparing by chi-square test. The relationship between PCSK9 levels and metabolic variables were performed by partial Spearman’s correlation analysis after adjusting for maternal age and gestational age. Logistic regression analysis was conducted to investigate the association between serum PCSK9 levels and the risk of incident GDM. A two-sided P value <0.05 was accepted as statistically significant. Sample size of 300 was evaluated according to a GDM prevalence of 11.91% in China (6). Referring to a recent study, the mean (SD) of PCSK9 in healthy populations was 283.68 (97.09) ng/ml (12). In our study, to detect a 50-ng/ml difference in PCSK9 values with a significance level of 0.05 between two groups, the power was 83.6% (α = 0.05).

## Results

### Baseline Characteristics of the Two Groups

The clinical and biochemical parameters of the groups were shown in **Table 1**. As we observed, maternal age, pre-body mass index (BMI), FPG, 1-h post-meal plasma glucose (1hPG), 2hPG, HbA1c, fasting insulin level (FINS), 1-h post-meal plasma insulin level (1hPINS), 2hPINS, HOMA-IR, obstetric history, uric acid, TG, white blood cell count, and circulating PCSK9 levels were much higher in the GDM group, while HDL and ISOGTT were significantly lower than those in the NGT group (all P < 0.05). There were no group differences in blood pressure, pregnancy BMI, parity times, abdominal perimeter, HOMA-β, LDL-C, TC, and Cr parameters (all P > 0.05) (**Table 1**).

**Table 1:** Clinical characteristics and circulating PCSK9 level of the groups studied.

Variable	GDM (n = 130)	NGT (n = 170)	P value
Age, years	30.01 ± 4.55	28.60 ± 5.00	0.021
Week of gestation, weeks	25.46 ± 1.01	25.29 ± 1.05	0.203
Parity times	2 (1–3)	2 (1–3)	0.058
Delivery times	0 (0–1)	0 (0–1)	0.301
Pre-BMI, kg/m^2^	22.82 ± 3.51	22.01 ± 2.84	0.045
BMI, kg/m^2^	25.05 ± 4.11	24.58 ± 2.86	0.539
Abdominal perimeter, cm	92.31 ± 7.98	91.08 ± 7.25	0.222
SBP, mm/Hg	115.17 ± 12.14	116.94 ± 10.63	0.241
DBP, mm/Hg	74.41 ± 9.01	73.31 ± 8.88	0.353
HbA1c (%)	5.16 ± 0.81	4.88 ± 0.32	<0.001
FPG, mmol/l	4.86 ± 0.57	4.45 ± 0.30	<0.001
1hPG, mmol/l	9.71 ± 1.55	7.37 ± 1.27	<0.001
2hPG, mmol/l	8.61 ± 1.53	6.43 ± 0.95	<0.001
FINS, mU/l	8.03 (4.95–10.94)	5.68 (3.42–8.65)	<0.001
1h PINS, mU/l	56.63 (41.87–81.31)	54.84 (34.11–70.20)	0.003
2h PINS, mU/l	64.62 (41.35–95.34)	54.35 (35.25–71.06)	0.004
HOMA-IR	1.69 (0.98–2.53)	1.05 (0.68–1.74)	<0.001
HOMA-β	122.72 (92.26–165.77)	129.63 (79.05–180.26)	0.987
ISOGTT	317.67 (260.43–410.01)	392.92 (310.45–503.10)	<0.001
ALT (U/L)	10 (7–16)	9 (6–17)	0.303
Cr (mmol/L)	39.29 ± 10.34	37.47 ± 7.74	0.130
Uric acid (U/L)	0.21 ± 0.06	0.19 ± 0.04	0.008
TC (mmol/L)	5.46 ± 0.87	5.43 ± 1.10	0.777
TG (mmol/L)	1.97 ± 1.03	1.68 ± 0.81	0.020
HDL (mmol/L)	2.61 ± 0.52	2.95 ± 0.62	0.000
LDL (mmol/L)	2.93 ± 0.69	2.90 ± 0.88	0.801
Family history of DM (%)	10%	1.43%	<0.001
PCSK9, ng/ml	268.07 ± 77.17	254.24 ± 74.22	0.001

Data are means ± standard deviation (SD) or medians (interquartile range).

pre-BMI, body mass index before pregnancy; BMI, body mass index in pregnancy; DBP, diastolic blood pressure; SBP, systolic blood pressure; FPG, fasting plasma glucose; 1hPG, 1-h post-meal plasma glucose; 2hPG, 2-h post-meal plasma glucose; FINS, fasting insulin level; 1hPINS, 1-h post-meal plasma insulin level; 2hPINS, 2-h post-meal plasma insulin level; ALT, alanine aminotransferase; Cr, serum creatinine; HbA1c, glycated hemoglobin; TC, total cholesterol; TG, triglyceride; HDL-C, high-density lipoprotein cholesterol; LDL-C, low-density lipoprotein cholesterol; HOMA-IR, homeostasis model assessment-insulin resistance; PCSK9, proprotein convertase subtilisin/kexin type 9.

### Correlations Between PCSK9 Levels and Metabolic Indices in GDM Group

As shown in **Table 2**, by partial Spearman correlation analysis, we found that serum PCSK9 levels were positively correlated with FPG (R = 0.251, P = 0.015), HbA1c (R = 0.275, P = 0.009), TC (R = 0.273, P = 0.010), and LDL-C (R = 0.326, P = 0.002) after adjustment of age and gestational age; we failed to observe any significant correlations between PCSK9 levels and other parameters (P > 0.05) in the GDM group.

**Table 2:** Partial Spearman correlations among PCSK9 and metabolic features in the GDM group.

Variable	R	P value
SBP	-0.03	0.816
DBP	0.09	0.460
Abdominal girth	0.022	0.841
BMI1	0.133	0.216
BMI2	0.04	0.724
FPG	0.251	0.015
1hPG	0.010	0.927
2hPG	0.118	0.271
HbA1c	0.275	0.009
FINS	0.021	0.827
1hPINS	0.067	0.483
2hPINS	-0.010	0.924
HOMA-IR	0.050	0.642
HOMA-β	-0.007	0.950
ISOGTT	-0.026	0.810
TC	0.273	0.010
HDL	-0.090	0.400
LDL	0.326	0.002
TG	0.121	0.260

Adjusted for age and gestational age.

pre-BMI, body mass index before pregnancy; BMI, body mass index in pregnancy; DBP, diastolic blood pressure; SBP, systolic blood pressure; FPG, fasting plasma glucose; 1hPG, 1-h post-meal plasma glucose; 2hPG, 2-h post-meal plasma glucose; FINS, fasting insulin level; 1hPINS, 1-h post-meal plasma insulin level; 2hPINS, 2-h post-meal plasma insulin level; ALT, alanine aminotransferase; Cr, serum creatinine; HbA1c, glycated hemoglobin; TC, total cholesterol; TG, triglyceride; HDL-C, high-density lipoprotein cholesterol; LDL-C, low-density lipoprotein cholesterol; HOMA-IR, homeostasis model assessment-insulin resistance; PCSK9, proprotein convertase subtilisin/kexin type 9.

### Associations of Circulating PCSK9 Levels With Risk of Incident Gestational Diabetes Mellitus

Binary logistic regression was carried out to assess the relationship between PCSK9 and the risk of GDM. The dependent variable was whether pregnant women were diagnosed with GDM, and the independent variables were age, gestational age, family history of diabetes, pre-BMI, abdominal girth, and PCSK9. The results were shown in **Table 3**. We observed that age [odds ratio (OR) = 5.412, P = 0.02] and serum PCSK9 levels (OR = 4.696, P = 0.03) were independently correlated with GDM.

**Table 3:** Logistic regression analysis on risk factors for gestational diabetes mellitus.

Variables	B	SE	OR value (95% CI)	P value
Age	0.064	0.030	1.066 (1.005–1.015)	0.032
Gestational age	0.153	0.140	1.165 (0.886–1.532)	0.286
Family history of diabetes	0.196	0.540	1.217 (0.423–3.503)	0.716
pre-BMI	0.043	0.067	1.044 (0.916–1.190)	0.519
Abdominal girth	0.008	0.029	1.008 (0.953–1.067)	0.773
PCSK9	0.009	0.003	1.009 (1.003–1.104)	0.003

pre-BMI, body mass index before pregnancy; CI, confidence interval; OR, odds ratio; SE, standard error; PCSK9, proprotein convertase subtilisin/kexin type 9.

We used logistic regression analysis model, taking the lowest PCSK9 quartile group (PCSK9 <223.94 ng/ml) as a reference, to further assume the prevalence of GDM according to quartiles of PCSK9. As presented in **Table 4**, the ORs for GDM were higher with increasing PCSK9 quartiles. In the highest PCSK9 quartile, the OR of GDM were 3.386 (95% CI 1.668–6.874, P = 0.001 for the trend). Furthermore, the upward trend remained even after adjustment of age, gestational age, BMI, blood pressure, abdominal girth, family history of diabetes mellitus, TG, LDL-C, and HOMA-IR (model 2, model 3, model 4) compared with those in the first quartile of PCSK9 (all P < 0.05 for a linear trend).

**Table 4:** Odds ratios and 95% confidence interval for GDM according to quartile of serum PCSK9 levels (n = 300).

	Q1	Q2	Q3	Q4	P value for trend
	OR (95% CI)	OR (95% CI)	OR (95% CI)	OR (95% CI)	
GDM					
Model 1	1	2.155 (1.071–4.337)	2.216 (1.093–4.493)	3.386 (1.668–6.874)	0.001
Model 2	1	2.517 (1.138–5.566)	2.520 (1.190–5.333)	3.818 (1.775–8.211)	0.001
Model 3	1	2.416 (1.098–5.312)	2.854 (1.241–6.564)	3.670 (1.652–8.155)	0.004
Model 4	1	2.337 (1.042–5.797)	3.068 (1.228–7.664)	3.559 (1.346–9.416)	0.009

Model 1 was not adjusted.

Model 2 was adjusted for age and gestational age.

Model 3 was adjusted for the variables in model 2 plus blood pressure, family history of diabetes mellitus, pre-BMI, and abdominal girth.

Model 4 was adjusted for the variables in model 3 plus TG, LDL-C, ALT, white blood cells, and HOMA-IR.

Subjects with a baseline circulating PCSK9 level in the lowest quartile group served as the reference group. Cutoff values in the four groups were Q1 <223.94 ng/ml, Q2 223.94–253.47 ng/ml, Q3 253.47–286.70 ng/ml, and Q4 >286.70 ng/ml.

GDM, gestational diabetes mellitus; OR, odds ratio; pre-BMI, body mass index before pregnancy; ALT, alanine aminotransferase; TG, triglyceride; LDL-C, low-density lipoprotein cholesterol; HOMA-IR, homeostasis model assessment-insulin resistance; PCSK9, proprotein convertase subtilisin/kexin type 9.

## Discussion

To our knowledge, very few studies have been performed to explore the status of PCSK9 in relation to metabolic factors in GDM subjects. Our data demonstrated that serum PCSK9 values were elevated significantly in the GDM group compared with those in the NGT group and correlated positively to HbA1c, LDL, TC, and FPG significantly. Moreover, a positive association was found between PCSK9 levels and the risk of GDM; the observation remained after adjustment of LDL-C and TG.

Early studies have reported that plasma PCSK9 values were elevated in T2DM patients (16–19). Moreover, PCSK9 is also increased in T1DM among younger subjects; with glycemic control worsening, plasma PCSK9 levels increased significantly (20). While Brouwers et al. (21) demonstrated that plasma PCSK9 was not altered in subjects with impaired glucose metabolism and T2DM. The findings were inconsistent. Notably, a recent study evaluated PCSK9 in GDM, finding no differences between GDM and healthy pregnant women (22). In our research, we found that serum PCSK9 levels were raised in GDM subjects as compared to those in NGT subjects. However, the underlying mechanism behind such elevation was unclear. It is reported that nutritional status and insulinemia modulate PCSK9 expression *via* a pathway involving sterol regulatory element-binding protein 1c (SREBP-1c) (23). Also, studies in adults coupled with studies in cells and mice indicated that hyperinsulinemia in obesity/T2DM might upregulate PCSK9 expression (24). In addition, a positive association was found between PCSK9 and insulin levels in a large pediatric population research (25). In view of these reports, insulinemia was an important factor influencing serum PCSK9 levels. Nevertheless, in this paper, we failed to find this observation. Early study reported that PCSK9 was increased in placentas from hypercholesterolemic pregnancies, presenting a protective role to prevent so much cholesterol transport from maternal to the fetal at the third trimester (26). However, our study was conducted in the second trimester of pregnancy; serum LDL-C levels were higher in GDM women, while the difference was not significant. Therefore, we considered that the effect of placenta in GDM on the remarkably increased serum PCSK9 levels was uncertain and may be slight in this period. More profound investigations are necessary to detect the PCSK9 expression of maternal blood, fetal blood, and placenta tissue in GDM subjects.

In line with previous data, our research also confirmed the significant positive relationships between PCSK9 and TC as well as LDL-C. On the other hand, accumulating data indicated that PCSK9 was associated with multiple metabolic factors including blood glucose, insulin concentration, HbA1c, and HOMA-IR (27–30). In this current study, we also found positive associations of PCSK9 with FPG and HbA1c, presenting a metabolic relationship between PCSK9 and GDM. But the exact mechanisms behind remain unclear. Recent study reported that PCSK9 was positively correlated with BMI in women and obesity was associated with elevated PCSK9 levels (29). Among the participants of our study, most GDM patients with a normal weight before pregnancy had a gestational weight gain within normal parameters in the second trimester of pregnancy and we failed to find a significant positive relationship between BMI and PCSK9 in the GDM group. Therefore, in our research, we think that BMI in this period may not have a notable effect on PCSK9 levels. As gestation proceeds, patients with GDM may end up on the verge of obesity in cases of progressive insulin resistance (IR), worsened glycemic control, excessive fat accumulation.

It is best known that PCSK9 binds to LDL-R, leading to their intracellular degradation and then promoting plasma LDL-C levels and hyperlipidemia. Previous evidence indicated that excessive cholesterol accumulation played a direct role in pancreatic islet dysfunction and might well be a key factor underlying the progression of diabetes (31). Prolonged exposure to high levels of LDL or very low-density lipoprotein (VLDL) could damage β-cell function and induce their necrosis (32, 33). Besides, published research indicated that PCSK9 was involved in inflammation (34). Li et al. (35) found that plasma PCSK9 levels were positively associated with the white blood cell count in coronary artery disease (CAD) patients. In our study, white blood cell count was higher in the GDM group while a significant positive association was not found. Further insight investigations are needed. T2DM coupled with a frequent status of lipid abnormalities is associated with an increased risk of CAD (36). PCSK9 inhibitor is a new class of drugs that markedly reduces plasma LDL-C levels, especially in combination with other lipid-lowering drugs. Hence, targeting PCSK9 represents an efficient therapeutic approach of improving diabetic dyslipidemia (37, 38). The inhibition of PCSK9 can be achieved by several approaches. Inclisiran is a novel gene silencing therapy of PCSK9 synthesis, lowering LDL-C levels and reducing the risk for CAD events (39, 40). However, PCSK9 mediates multifarious functions instead of well-known functions of lipid metabolism regulation. The long-term safety of targeting PCSK9 is still unknown. Additionally, antibodies that inhibit PCSK9 should not be used in children and pregnant populations because of their unwarrantable safety (41).

In the present study, we discovered that serum PCSK9 levels showed a positive association with the risk of GDM from the data during the second trimester of pregnancy independent of several potential factors.

Although available data from early epidemiological and clinical trials documented that serum PCSK9 levels increased the incidence risk of DM, genetic findings were completely opposite. Therefore, the link between PCSK9 and DM was still conflicting and controversial (42). Further intensive studies are required to prove the cause-and-effect relationship between the two.

Several limitations need to be acknowledged. First, the study was unable to establish causality due to its observational nature. Second, we used HOMA-IR formula but not the standard method to precisely evaluate the degree of insulin resistance. Third, we failed to observe long-term changes of PCSK9 levels throughout pregnancy and after delivery.

## Conclusions

In summary, this current study found that circulating PCSK9 levels increased significantly in the GDM group with a close relation to LDL-C, FPG, and HbA1c. Furthermore, serum PCSK9 levels were positively associated with the risk of GDM, suggesting a possible link between PCSK9 and GDM.

## Data Availability Statement

The original contributions presented in the study are included in the article/supplementary material. Further inquiries can be directed to the corresponding authors.

## Ethics Statement

The study was approved by the Ethics Committee of the Xinhua Hospital Affiliated to Shanghai Jiao Tong University School of Medicine, and all participants signed an informed consent form. The patients/participants provided their written informed consent to participate in this study.

## Author Contributions

LQ defined the research theme. YW, JS, and ZY performed the experiments, collected and analyzed the data, and wrote the paper. QS revised the article for important intellectual content. All authors contributed to the article and approved the submitted version.

## Funding

This work was supported by the Shanghai Municipal and Health Commission project (20204Y0294), College-level topics of Xinhua Hospital Chongming Branch Affiliated to Shanghai Jiao Tong University School of Medicine.

## Conflict of Interest

The authors declare that the research was conducted in the absence of any commercial or financial relationships that could be construed as a potential conflict of interest.

## Publisher’s Note

All claims expressed in this article are solely those of the authors and do not necessarily represent those of their affiliated organizations, or those of the publisher, the editors and the reviewers. Any product that may be evaluated in this article, or claim that may be made by its manufacturer, is not guaranteed or endorsed by the publisher.
